# PPAR-Mediated Bile Acid Glucuronidation: Therapeutic Targets for the Treatment of Cholestatic Liver Diseases

**DOI:** 10.3390/cells13151296

**Published:** 2024-08-01

**Authors:** Gina M. Gallucci, Colleen M. Hayes, James L. Boyer, Olivier Barbier, David N. Assis, Nisanne S. Ghonem

**Affiliations:** 1 Department of Biomedical and Pharmaceutical Sciences, College of Pharmacy, University of Rhode Island, Kingston, RI 02881, USA; 2 Section of Digestive Diseases, Yale School of Medicine, New Haven, CT 06510, USA; 3 Faculty of Pharmacy, Laval University, Québec, QC G1V 0A6, Canada

**Keywords:** primary biliary cholangitis, primary sclerosing cholangitis, cholestasis, peroxisome proliferator-activated receptor, bile acid glucuronidation

## Abstract

Cholestatic liver diseases, including primary biliary cholangitis (PBC) and primary sclerosing cholangitis (PSC), result from an impairment of bile flow that leads to the hepatic retention of bile acids, causing liver injury. Until recently, the only approved treatments for PBC were ursodeoxycholic acid (UDCA) and obeticholic acid (OCA). While these therapies slow the progression of PBC in the early stage of the disease, approximately 40% of patients respond incompletely to UDCA, and advanced cases do not respond. UDCA does not improve survival in patients with PSC, and patients often have dose-limiting pruritus reactions to OCA. Left untreated, these diseases can progress to fibrosis and cirrhosis, resulting in liver failure and the need for transplantation. These shortcomings emphasize the urgent need for alternative treatment strategies. Recently, nuclear hormone receptors have been explored as pharmacological targets for adjunct therapy because they regulate enzymes involved in bile acid metabolism and detoxification. In particular, the peroxisome proliferator-activated receptor (PPAR) has emerged as a therapeutic target for patients with PBC or PSC who experience an incomplete response to UDCA. PPARα is predominantly expressed in the liver, and it plays an essential role in the regulation of cytochrome P450 (CYP) and uridine 5’-diphospho-glucuronosyltransferase (UGT) enzymes, both of which are critical enzyme families involved in the regulation of bile acid metabolism and glucuronidation, respectively. Importantly, PPARα agonists, e.g., fenofibrate, have shown therapeutic benefits in reducing elevated markers of cholestasis in patients with PBC and PSC, and elafibranor, the first PPAR (dual α, β/δ) agonist, has been FDA-approved for the second-line treatment of PBC. Additionally, newer PPAR agonists that target various PPAR isoforms (β/δ, γ) are under development as an adjunct therapy for PBC or PSC, although their impact on glucuronidation pathways are less characterized. This review will focus on PPAR-mediated bile acid glucuronidation as a therapeutic pathway to improve outcomes for patients with PBC and PSC.

## 1. Introduction

Bile acid synthesis and secretion are essential functions of the human liver. Bile acids are critical to maintaining cholesterol and metabolic homeostasis [1]. However, when elevated, bile acids become cytotoxic molecules that can lead to inflammation, fibrosis, cirrhosis, and eventually liver failure. Primary biliary cholangitis (PBC) and primary sclerosing cholangitis (PSC) are cholestatic liver diseases characterized by the accumulation of bile acids in the liver [2]. Ursodeoxycholic acid (UDCA) is the first line of treatment for patients with PBC; however, ~40% of patients have an incomplete response [3] and it has not been shown to improve survival in PSC [4]. Obeticholic acid (OCA) is also FDA-approved for the treatment of PBC; however, its anti-cholestatic effect is modest and pruritus is a limiting side effect [5]. Due to these therapeutic shortcomings, alternative therapies are needed to reduce bile acid-induced liver injury and slow the progression of liver injury.

The peroxisome proliferator-activated receptor (PPAR), a ligand-activated transcription factor of the nuclear receptor superfamily, includes three distinct isoforms (α, β/δ, and γ) that are encoded by different genes [6,7,8]. PPAR is an important regulator of several genes involved in bile acid synthesis and metabolism [9]. Upon ligand binding, PPAR forms a heterodimer with the retinoid x receptor (RXR) and binds to PPAR response elements (PPREs) in target genes to regulate gene transcription. PPREs are characterized by direct repeats of AGGTCA spaced by one nucleotide [10]. PPARα is expressed in tissues that undergo high fatty acid catabolism, e.g., the liver, kidney, intestine, and brown adipose tissue [11], where it also stimulates fatty acid catabolism [12] and attenuates inflammatory responses [13]. PPARα ligands include endogenous molecules, e.g., fatty acids and arachidonic acid derivatives, the synthetic agonist Wy-14,643, and the FDA-approved hypolipidemic drug, fenofibrate. Importantly, adjunct therapy with fibrates, e.g., fenofibrate and bezafibrate (pan-PPAR agonist; not FDA-approved), improves biochemical markers of cholestasis, including reducing elevated serum alkaline phosphatase (ALP) and reducing the toxicity of the bile acid pool in PBC and PSC [14,15,16,17,18,19]. Based on these findings, the role of PPAR(α) in bile acid detoxification, specifically in regulating the phase II metabolic pathway of bile acid glucuronidation, has emerged as an area of therapeutic interest to improve the treatment of cholestatic liver diseases. 

Glucuronidation is one of the main mechanisms of elimination, e.g., a detoxification process, of endogenous substances and xenobiotics in humans [20]. Glucuronidation is an enzymatic reaction catalyzed by the family of uridine 5’-diphospho-glucuronosyltransferase (UGT) enzymes, which involves the conjugation of glucuronic acid to the 3/6-hydroxyl or 24-carboxyl bile acid steroid nucleus, to generate -3/-6 or -24 bile acid glucuronides (3/6/24-G). In general, the glucuronidation reaction enhances the hydrophilicity of the molecules, making bile acids more readily excreted [21], thereby reducing bile acid-induced liver injury and facilitating bile acid detoxification. Evidence indicates that bile acid glucuronidation is an increasingly important and active process under cholestatic conditions [22,23]. Importantly, PPARα transactivates several UGT enzymes involved in bile acid glucuronidation, suggesting this nuclear receptor could be a therapeutic target to promote bile acid detoxification to treat cholestatic liver diseases. This review will focus on PPAR-mediated bile acid glucuronidation to reduce bile acid-induced liver injury and improve outcomes for patients with refractory cholestatic liver diseases. 

## 2. Methods

Literature searches were conducted in databases such as PubMed and Google Scholar. Search terms including “bile acid glucuronidation”, “PPARα”, “cholestasis”, “primary biliary cholangitis”, “primary sclerosing cholangitis”, “bile acid synthesis”, and “treatments for cholestasis” were used. The primary topics of interest were bile acid metabolism, bile acid glucuronidation, and PPAR agonists for treating cholestatic liver diseases. Articles that did not describe clinical outcomes, bile acid metabolism, or detoxification in relation to cholestatic liver diseases were excluded. 

## 3. Bile Acid Synthesis and Regulation 

Bile acid synthesis is critical to maintaining liver cholesterol homeostasis (reviewed in [22]). In a healthy adult, approximately 0.2–0.6 g of bile acids are synthesized daily from cholesterol in the liver, and the daily net turnover of bile acids is ~3 g, or ~5% of the total bile acid pool [24]. The two major pathways responsible for bile acid synthesis are the classic (or neutral) and the alternative (or acidic). The classic pathway is the major route of bile acid synthesis in humans, and it is initiated by cytochrome P450 7A1 (CYP7A1), the rate-limiting enzyme in the pathway. During classical bile acid synthesis, cholesterol is metabolized to 7α-hydroxycholesterol by CYP7A1, and hydroxysteroid dehydrogenase 3B7 converts 7α-hydroxycholesterol to 7α-hydroxy-4-cholesten-3-one (C4). Next, C4, a surrogate serum marker of bile acid synthesis [25], can be converted to the primary bile acid cholic acid (CA) by CYP8B1 or to chenodeoxycholic acid (CDCA) [26]. 

In the alternative pathway, cholesterol is converted to 25(R)-26-hydroxycholesterol or 25-hydroxycholesterol by CYP27A1 [27] or CYP3A4 [28], respectively, which undergo hydroxylation at the 7α-hydroxy steroid nucleus by CYP7B1 for the subsequent synthesis of CA and CDCA. Next, CA and CDCA, produced in the liver, are converted to secondary bile acids, e.g., deoxycholic acid (DCA) and lithocholic acid (LCA), by bacterial 7α-dehydroxylating enzymes present in the intestinal microflora [29]. However, these secondary bile acids are more hydrophobic, e.g., cytotoxic, and elevated levels of these hydrophobic bile acids have been associated with liver carcinogenesis [30] and hepatotoxicity [31] in mice. Approximately 95% of the bile acids in the intestine are reabsorbed and transported back to the liver via the portal vein [32]. Once reabsorbed, the remaining secondary bile acids can undergo hydroxylation at the 6-hydroxyl steroid nucleus by CYP3A4 to hyodeoxycholic acid (HDCA) and hyocholic acid (HCA) [33]. Furthermore, most bile acids in humans can also be conjugated to glycine and taurine, at a ~3:1 ratio [34]. Newer evidence indicates that organisms within the human microbiome can synthesize unique amino acid amide conjugates to CA, namely phenylalanine, tyrosine, or leucine, to produce microbially conjugated bile acids, including phenylalanocholic acid, tyrosocholic acid, and leucocholic acid [35]. Under healthy conditions, bile acid synthesis is essential to maintain lipid and cholesterol homeostasis; however, when the process is dysregulated, the accumulation of bile acids in the liver can lead to oxidative stress and apoptosis and the development of cholestatic liver diseases [36]. 

## 4. Bile Acid Detoxification: Glucuronidation

Glucuronidation is an important phase II metabolic pathway involved in the detoxification of numerous endogenous compounds including bile acids, steroids, and xenobiotics [23,37]. In humans, hydrophobic molecules, e.g., bile acids, undergo glucuronidation catalyzed by UGT enzymes [38]. In healthy individuals, bile acid glucuronides represent approximately 10% of the total serum bile acid pool [39] and ~12–36% of the total bile acids excreted in urine daily [40]. Within the liver, bile acid glucuronidation is catalyzed by the UGT1A and 2B families, particularly UGT1A3 [41], UGT1A4 [39], UGT2B4 [42], and UGT2B7 [41,42], and each UGT is responsible for the conjugation of parent bile acids [21]. Although additional UGT isoforms including 2A1 and 2A2 have been identified as bile acid-glucuronidating enzymes [43], their expression in the human liver has not been observed [44,45]. 

**Figure 1:**
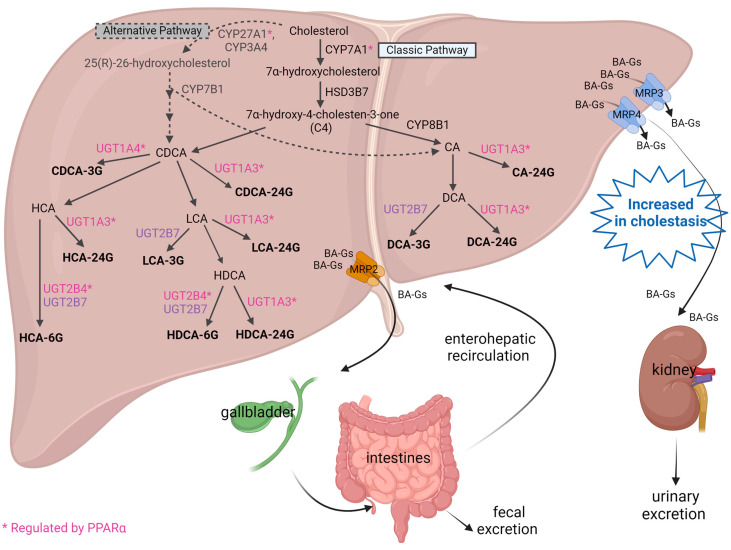
Two major bile acid biosynthetic pathways are shown. In the classic pathway, cytochrome P450 (CYP)7A1 converts cholesterol to 7α-hydroxycholesterol, which is converted to 7α-hydroxy-4-cholesten-3-one (C4) by 3*β*-hydroxysteroid dehydrogenase (HSD3B7). C4 is converted to cholic acid (CA) by CYP8B1 or to chenodeoxycholic acid (CDCA). In the alternative pathway, CYP27A1 or CYP3A4 converts cholesterol to 25(R)-26-hydroxycholesterol, followed by hydroxylation by CYP7B1 for the eventual synthesis of CA and CDCA. CA is converted to deoxycholic acid (DCA), and CDCA can be converted to lithocholic acid (LCA) or hyocholic acid (HCA). LCA can be hydroxylated to form hyodeoxycholic acid (HDCA). Uridine 5’-diphospho-glucuronosyltransferase (UGT) enzymes catalyze the glucuronidation of these bile acids to form -3, -6, or -24-glucuronides (-G). Bile acid glucuronides (BA-Gs) are transported out of the liver and into the bile canalicular space via the multidrug resistance-associated protein (MRP)-2 and pass through the common bile duct, gallbladder, and intestines, where they can be recycled back to the liver via enterohepatic recirculation. Alternatively, BA-Gs can be transported out of the liver and into systemic circulation via MRP3 and MRP4 for eventual renal excretion into urine. Image created with BioRender.com.

In general, glucuronidation enhances the negative charge of the bile acid, thereby facilitating its export out of the liver and into systemic circulation for subsequent renal excretion [46], i.e., bile acid detoxification. Specifically, glucuronidation involves the transfer of the glucuronosyl group from uridine 5-diphosphoglucuronic acid (UDPGA) to acceptor substrates, including the 3/6-hydroxyl or 24-carboxyl bile acid steroid nucleus, to produce corresponding ether -3/6G or acyl -24G [47,48,49]. Hyocholic acid (HCA)-6G and hyodeoxycholic acid (HDCA)-6G are among the most abundant bile acid glucuronides found in the serum of non-cholestatic adults [39], patients with PBC and PSC [18], and in the urine of adults with biliary obstruction, pre- and post-biliary stenting [48]. Based on its role as a detoxification pathway, bile acid glucuronidation is an increasingly important elimination mechanism to reduce bile acid-induced liver injury during cholestasis. A schematic representation of bile acid metabolism and glucuronidation in the liver is presented in Figure 1.

## 5. PPAR Agonists Regulate Bile Acid Glucuronidation

In the past decade, PPAR has emerged as a therapeutic target to treat cholestatic liver diseases due to its regulation of the multi-enzymatic process of bile acid synthesis and metabolism. In particular, PPARα downregulates bile acid synthesis by inhibiting CYP enzymes, e.g., CYP7A1 and CYP27A1 [50], making it a desirable target for the pharmacological treatment of cholestatic liver diseases. It has been well documented that PPARα activators suppress CYP7A1 gene promoter activity in human hepatoma HepG2 cells [51,52] and that PPARα agonists, e.g., fenofibrate, downregulate cyp7a1 and cyp27a1 expression and reduce their activity in rodents [53]. Regarding bile acid levels, fenofibrate significantly reduced serum levels of CDCA and the secondary bile acids DCA, LCA, and HDCA, in non-cholestatic adult volunteers [54]. By decreasing the levels of the hydrophobic bile acids DCA and LCA, fenofibrate therapy reduces the concentration of the toxic bile acid pool. Likewise, Ghonem et al. [14] showed that patients with PBC and PSC who received combination treatment with UDCA and fenofibrate have decreased serum levels of total, primary, secondary, glycine-, and taurine-conjugated bile acids. These clinical data demonstrate that combination treatment with UDCA and fenofibrate contributes to the detoxification of the serum bile acid pool in refractory cholestasis. 

**Figure 2:**
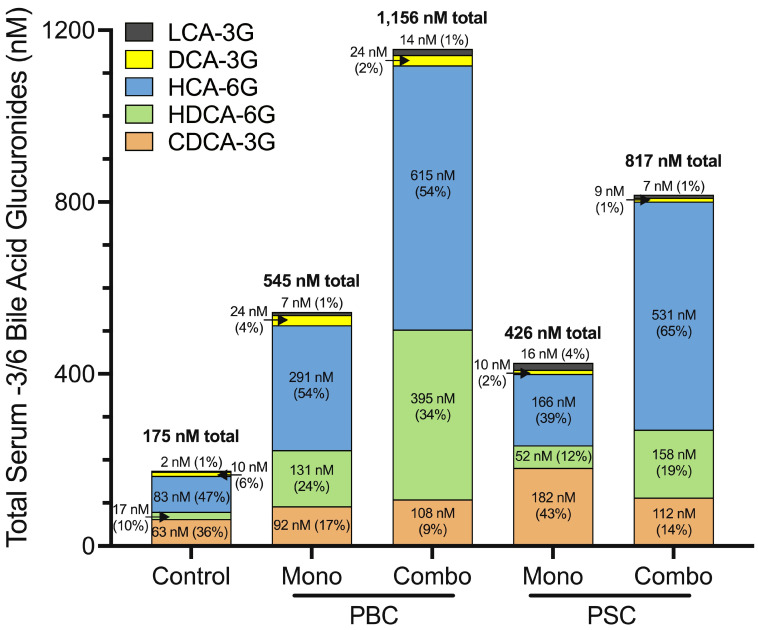
Combination treatment with UDCA and fenofibrate alters the concentration and composition of -3/6 bile acid glucuronides during cholestatic liver diseases. Serum was collected from patients treated with either UDCA monotherapy (13–15 mg/kg/day)—“mono”—or combination treatment with UDCA and fenofibrate (145–160 mg/day)—“combo”. Serum bile acid glucuronide concentrations were measured using LC-MS/MS methodology. Data represent the total concentration of -3 and -6 bile acid glucuronides within each patient cohort [18]. PBC (*n* = 16 “mono”, *n* = 16 “combo”) and PSC (*n* = 11 “mono”, *n* = 12 “combo”). UDCA: ursodeoxycholic acid; PBC: primary biliary cholangitis; PSC: primary sclerosing cholangitis; LCA: lithocholic acid; DCA: deoxycholic acid; HCA: hyocholic acid; HDCA: hyodeoxycholic acid; CDCA: chenodeoxycholic acid.

In addition to regulating CYP enzymes involved in bile acid synthesis, PPARα directly activates several UGT enzymes [42,55] involved in bile acid and bilirubin glucuronidation to promote the production of and enhance the urinary excretion of glucuronide conjugates. We have shown that adjunct fenofibrate therapy shifts the composition of the total bile acid pool to be less toxic, compared to ongoing cholestasis while receiving UDCA monotherapy [14,18], and also increases total serum bile acid glucuronides by ~2-fold vs. UDCA monotherapy and ~6-fold compared to healthy controls [18]. However, not all bile acid glucuronides are non-toxic; some bile acid glucuronide species exert cytotoxic properties, making their accumulation harmful. For example, HepG2 cells treated with CDCA-3G and DCA-3G undergo apoptosis and necrosis [56], and LCA-3G induces cholestasis in rats [57], suggesting that an increase in total serum bile acid glucuronide levels is not an adequate indicator of therapeutic response. Rather, the individual bile acid glucuronide species are important determinants of therapeutic benefit. Indeed, serum levels of toxic glucuronides, e.g., CDCA-, DCA-, and LCA-3G, correlate with elevated serum ALP levels in patients with PBC and PSC who have an incomplete response to UDCA monotherapy. However, add-on fenofibrate reduces the percentage of toxic serum LCA-3G, DCA-3G, and CDCA-3G levels, while increasing the proportion of non-toxic species, e.g., HCA-6G and HDCA-6G, within the total bile acid glucuronide pool, presented in Figure 2 [18].

Thus, shifting the composition of total bile acids in favor of the selective non-toxic bile acid glucuronides points towards a mechanistic target to reduce bile acid-induced liver injury. Therefore, bile acid glucuronidation may be a pathway of interest to regulate bile acid detoxification during cholestasis pharmacologically. Below, we review multiple studies that have evaluated the role of PPARα in regulating UGT enzymes; only the UGT enzymes related to bilirubin and bile acid glucuronidation in the liver will be discussed, namely UGT1A1, 1A3, 1A4, 2B4, and 2B7.

***UGT1A1*:** UGT1A1 (along with UGT1A4) is responsible for the glucuronidation of bilirubin [58,59,60,61]. Bilirubin glucuronidation is the rate-limiting step in bilirubin excretion [62], and hyperbilirubinemia is a characteristic found in individuals with cholestasis [63]. Rats treated with PPARα agonists showed increased hepatic UGT1A1 mRNA [64], and PPARα activation by clofibrate in primary rat hepatocytes increased UGT1A1 protein expression [64,65]. Additionally, UGT1 transgenic mice treated with Wy-14,643 showed increased UGT1A1 mRNA and protein expression, and a functional PPRE was identified in the UGT1A1 promoter [55]. These effects were observed in rats treated with ciprofibrate, as PPARα activation-induced bilirubin glucuronidation activity [66]. We [18] and others [55,67] have shown that fenofibrate and Wy-14,643 upregulate UGT1A1 mRNA levels in both HepG2 cells and primary human hepatocytes. Importantly, patients with PBC and PSC who receive a combination treatment with UDCA and fenofibrate show significantly reduced elevated levels of serum total bilirubin levels [14], suggesting that the activation of PPARα may reduce bilirubin levels through UGT1A1 enzyme activation.

***UGT1A3:*** UGT1A3 is responsible for the formation of 24-G, e.g., CA-, CDCA-, DCA-, LCA-, HCA-, and HDCA-24G [21,41,49]. Fenofibrate and Wy-14,643 stimulate UGT1A3 expression [39], and treatment with Wy-14,643 increases CDCA-24G formation in human hepatocytes and HepG2 cells [49]. Additionally, UGT1A3 mRNA was upregulated in UGT1A transgenic mice treated with Wy-14,643, and a functional PPRE was identified in the UGT1A3 promoter [55]. In non-cholestatic volunteers [39] and cholestatic patients [14], fenofibrate treatment increased serum levels of some bile acid-24 glucuronides, compared to no treatment and UDCA treatment, suggesting that PPARα activation promotes their production through the transcriptional regulation of UGT1A3. However, it is unknown whether the (low) abundance of bile acid acyl glucuronides detected in human serum [18,39] was the result of preferential glycine- and taurine-conjugation to the C_24_ carboxyl group [68], or as a result of degradation due to temperature and pH instability [47], in which case, serum levels of these bile acid acyl glucuronides may be underestimated.

***UGT1A4*:** UGT1A4 contributes to the production of bilirubin glucuronide and CDCA-3G. PPARα activation by fenofibrate and Wy-14,643 induced UGT1A4 expression in primary human hepatocytes [18,55] and HepG2 cells [39]. However, serum CDCA-3G levels decreased in patients with PBC and PSC after combination treatment with fenofibrate and UDCA [18], suggesting the enhanced secretion of CDCA-3G. Patients with biliary obstruction showed elevated serum CDCA-3G levels, which were greatly reduced when bile flow was restored, suggesting that UGT enzymes and/or bile acid glucuronide transporters may be altered when there is an obstruction to bile flow [48].

***UGT2B4:*** UGT2B4 (and UGT2B7) are responsible for the formation of HDCA-6G [42,69] and HCA-6G [21]. Barbier et al. identified a PPARα response element within the UGT2B4 promoter of HepG2 cells [42]. In primary human hepatocytes, PPARα activation by fenofibrate [18] and Wy-14,643 upregulates UGT2B4 mRNA expression [39]. Treatment with fenofibrate also increases the serum levels of HDCA-6G and HCA-6G in non-cholestatic patients [39] and patients with PBC and PSC [18]. In addition, Wy-14,643 treatment induced UGT2B4 mRNA expression in HepG2 cells, and the combination treatment of Wy-14,643 and CDCA, a Farnesoid X receptor (FXR) agonist, induced UGT2B4 mRNA by more than 5 times that of Wy-14,643 treatment alone, indicating that FXR and PPARα may cooperatively regulate UGT2B4 expression [42]. Interestingly, the serum levels of HDCA-6G were also increased in patients with biliary obstruction after biliary stenting [48]. Thus, the observed increases in HDCA- and HCA-6G may result from the dual activation of FXR and PPARα in cholestatic patients treated with UDCA and fenofibrate.

***UGT2B7*:** UGT2B7 contributes to the formation of -3 and -6 bile acid glucuronides [39,42] and has recently been reported as one of the most relevant UGT enzymes in bile acid glucuronidation due to its production of HDCA-G in human recombinant enzyme and human liver microsomes [70]. In primary human hepatocytes, however, neither fenofibrate nor Wy-14,643 increased UGT2B7 mRNA expression [39].

In line with the impact of bile acid and bile acid glucuronide composition, single-nucleotide polymorphisms (SNPs) in UGT1A3 and 2B genes have been shown to alter the production of bile acid glucuronides in healthy volunteers [39], whereas other SNPs, e.g., UGT1A1, influence patient outcomes in PSC [71]. As such, the impact of UGT1A and 2B polymorphisms on treatment response to PPAR-mediated BA glucuronidation during cholestasis is unknown and should be further studied to understand their therapeutic impact during cholestasis.

In summary, the data presented above support the notion that PPARα activation induces UGT1A1, 1A3, 1A4, and 2B4 expression, leading to a therapeutic bile acid glucuronidation profile and favorable clinical outcomes for patients experiencing a sub-therapeutic response to UDCA. Additional data including urinary bile acid glucuronide levels, particularly the -3 and -6 glucuronides, are needed from cholestatic patients to investigate further the mechanisms of PPARα-mediated bile acid detoxification. 

## 6. New PPAR Agonists under Investigation for the Treatment of Cholestatic Liver Diseases 

Recently, newer PPAR agonists have been developed with higher potencies for PPAR isoforms (α, β/δ, and γ), including seladelpar (PPAR β/δ), elafibranor (dual PPARα, β/δ), and saroglitazar (dual PPARα, γ). These newer PPAR agonists are under clinical investigation for second-line treatment of PBC and for first-line treatment of PSC, of which elafibranor was FDA-approved for PBC in June 2024. Despite the recent interest in additional PPAR isoforms (β/δ, γ) to treat cholestatic liver diseases, the roles of these additional PPAR isoforms in regulating the bile acid metabolism pathway are less characterized, although some studies have begun to elucidate this pathway. In studies with primary human and mouse hepatocytes, treatment with seladelpar significantly decreased CYP7A1 mRNA expression, and cyp7a1 mRNA and protein expression, respectively [72]. Furthermore, seladelpar treatment decreased the hepatic mRNA expression of cyp7a1 and plasma levels of C4 in wild-type mice [72] and hepatic cyp7a1 gene expression in diet-induced obese non-alcoholic steatohepatitis (NASH) mice [73]. Similarly, elafibranor significantly decreased hepatic cyp7a1 expression and inflammatory markers in a hamster model of NASH and heart failure [74]. In addition, elafibranor treatment reduced cholate, chenodeoxycholate, and ursodeoxycholate levels in a diet-induced obese mouse model of non-alcoholic fatty liver disease [75]. Therefore, the activation of PPARα and β/δ in combination or alone is associated with the downregulation of bile acid synthesis, and each isoform has unique effects on the bile acid pool. Interestingly, treatment with MBT1805, a pan-PPAR agonist, significantly reduced the expression of cyp27A1 and elevated levels of taurine- and glycine-conjugated bile acids in an ANIT-induced cholestatic mouse model [76]. Similarly, the pan-PPAR agonist bezafibrate decreased the mRNA expression of CYP7A1 and CYP27A1 in HepaRG cells [16] and decreased hepatic CYP7A1 activity by nearly 60% in patients with gallstones undergoing cholecystectomy [77]. In the BEZURSO phase 2 clinical trial, the proportion of endogenous bile acids within the bile acid pool and C4 levels decreased in bezafibrate-treated patients [19]. Clinical trials of elafibranor [78] and seladelpar [79,80] have also shown decreases in circulating levels of C4, along with significant decreases in total bile acids from seladelpar treatment [81]. These data demonstrate that the activation of PPAR therapeutically reduces bile acid toxicity and supports newer PPAR agonists targeting specific PPAR isoforms to treat cholestasis.

It should be acknowledged that PPAR therapy in cholestasis has several potential limitations. A small percentage of patients can develop myositis on fibrate therapy, and, therefore, some practitioners measure serum creatine kinase following the addition of fibrates. However, clinical trials of newer PPAR agents including seladelpar, elafibranor, and saroglitazar did not show a significant increase in myositis nor other relevant safety signals. More importantly, while clinical studies in PBC demonstrate a statistically significant biochemical response to PPAR agents, a sizeable percentage of patients do not respond to this modality. The reasons for this are unknown at present, leading to early trials in PBC of triple therapy with UDCA, PPAR agonists, and OCA. It remains to be determined whether UGT polymorphisms or other aspects influencing the ability of PPAR agonists to achieve bile acid glucuronidation are the cause of non-response to PPAR treatment and, therefore, this possibility represents an appropriate topic for further studies.

## 7. Conclusions

This review has provided an up-to-date summary of the contribution of PPAR activation to bile acid glucuronidation, specifically the role of PPARα in reducing bile acid toxicity for patients with persistent cholestatic liver injury. We have compiled the published evidence to date that supports the PPARα activation of UGT1A1, 1A3, and 2B4 and how this activation contributes to the formation of bile acid glucuronides that are then renally excreted, as shown in Figure 1. We have also provided a summary of the additional PPAR agonists’ roles in regulating bile acid metabolism; these newer agonists are currently in clinical development for the treatment of cholestatic liver diseases, including elafibranor, the first PPAR agonist FDA-approved for second-line treatment of PBC. While not covered in this review, it is important to mention that other bile acid detoxification pathways, e.g., sulfonation, also promote the detoxification of the bile acid pool through PPAR-mediated mechanisms [82]. Nevertheless, the bile acid glucuronidation pathway is of high clinical interest. The role of bile acid glucuronidation and potential UGT polymorphisms in cholestatic patients is an evolving field that warrants further investigation to understand how these patients may be treated in the future.

## Data Availability

Not applicable.
